# Five-wave-packet quantum error correction based on continuous-variable cluster entanglement

**DOI:** 10.1038/srep15462

**Published:** 2015-10-26

**Authors:** Shuhong Hao, Xiaolong Su, Caixing Tian, Changde Xie, Kunchi Peng

**Affiliations:** 1 State Key Laboratory of Quantum Optics and Quantum Optics Devices, Institute of Opto-Electronics, Shanxi University, Taiyuan 030006, P. R. China

## Abstract

Quantum error correction protects the quantum state against noise and decoherence in quantum communication and quantum computation, which enables one to perform fault-torrent quantum information processing. We experimentally demonstrate a quantum error correction scheme with a five-wave-packet code against a single stochastic error, the original theoretical model of which was firstly proposed by S. L. Braunstein and T. A. Walker. Five submodes of a continuous variable cluster entangled state of light are used for five encoding channels. Especially, in our encoding scheme the information of the input state is only distributed on three of the five channels and thus any error appearing in the remained two channels never affects the output state, i.e. the output quantum state is immune from the error in the two channels. The stochastic error on a single channel is corrected for both vacuum and squeezed input states and the achieved fidelities of the output states are beyond the corresponding classical limit.

The transmission of quantum states with high fidelity is an essential requirement for implementing quantum information processing with high quality. However, losses and noises in channels inevitably lead to errors into transmitted quantum states and thus make the distortion of resultant states. The aim of quantum error correction (QEC) is to eliminate or, at least, reduce the hazards resulting from the imperfect channels and to ensure transmission of quantum states with high fidelity^1^. A variety of discrete variable QEC protocols, such as nine-qubit code^2^, five-qubit code^3^, topological code^4^,^5^, have been suggested and the experiments of QEC have been realized in different physical systems, such as nuclear magnetic resonance^6^,^7^,^8^, ionic^9^,^10^, photonic^11^,^12^, superconducting systems^13^,^14^ and Rydberg atoms^15^.

Besides quantum information with discrete variables, quantum information with continuous variables (CV) is also promptly developing^16^,^17^,^18^,^19^,^20^,^21^,^22^,^23^. Different types of CV QEC codes for correcting single non-Gaussian error have been proposed, such as nine-wave-packet code^24^,^25^, five-wave-packet code^26^,^27^, entanglement-assisted code^28^ and erasure-correcting code^29^. A CV QEC scheme against Gaussian noise with a non-Gaussian operation of photon counting has been also theoretically analyzed^30^. The CV QEC schemes of the nine-wave-packet code^31^, erasure-correcting code against photon loss^32^ and the correcting code with the correlated noisy channels^33^ have been experimentally demonstrated.

According to the no-go theorem proved in ref. 34, Gaussian errors are impossible to be corrected with pure Gaussian operations. However, non-Gaussian stochastic errors, which frequently occur in free-space channels with atmospheric fluctuations for example^35^,^36^,^37^, can be corrected by Gaussian schemes since the no-go theorem does not apply in this case. Generally, the stochastic error model is described by^38^


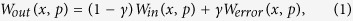


where the input state *W*_*in*_(*x*, *p*) is transformed into a new state *W*_*error*_(*x*, *p*) with probability *γ* or it remains unchanged with probability 1 − *γ*. Even for the case of two Gaussian states *W*_*in*_(*x*, *p*) and *W*_*error*_(*x*, *p*), the output state *W*_*out*_(*x*, *p*) is also non-Gaussian, that is, this channel model describes a certain, simple form of non-Gaussian errors.

In 2009 T. Aoki *et al*. presented the first experimental implementation of a Shor-type nine-channel QEC code based on entanglement among nine optical beams, which was the achievable largest entangled state on experiments then^31^. This scheme is deterministically implemented using only linear operations and resources, which can correct arbitrary single beam error. Although S. L. Braunstein discovered a highly efficient five-wave-packet code theoretically in 1998, its linear optical construction was not proposed^26^. Later, in 2010, T. A. Walker and S. L. Braunstein outlined a new approach for generating linear optics circuits that encode QEC code and proposed a linear optics construction for a five-wave-packet QEC code^27^. Differentiating from previous approaches by means of directly transferring existing qubit codes into CV codes, they defined the conditions for yielding a CV QEC code firstly and then searched numerically for circuits satisfying this criterion. The five-wave-packet code improves on the capacity of the best known code implemented by linear optics and saturates the lower bound for the number of carrier needed for a single-error-correct code^27^. However, the proposed five-wave-packet CV QEC code has not been experimentally demonstrated so far.

Based on the approach outlined by T. A. Walker and S. L. Braunstein^27^, we design a more compact linear optics construction and achieve the first experimental demonstration of five-wave-packet CV QEC code using a five-partite CV cluster entangled state^39^,^40^. In this experiment only four ancilla squeezed states of light are required and four optical beamsplitters are used in the encoding and the decoding system, respectively. Comparing with the nine-wave-packet system^31^, the required quantum resources and utilized optical elements in our system decrease a half. The smaller codes not only save quantum resources, but also increase data rates and decrease the chance of further occurring errors, thus are very significant for the development of quantum information technology^27^. In the presented encoding method, only a part of all wave packets (three of five in the presented experiment) involves the information of the input state and therefore the noise occurring in the remained channels (channels 1 and 2 in the presented system) do not introduce any error into the transmitted quantum state. Such that, we do not need to perform the error correction for the remained channels and the near unity fidelity is achieved in these channels. We name the encoding method as the partial encoding. It should be emphasized that although the remained two channels do not involve the information of the input state, they play the unabsolvable roles in the syndrome recognition and the error correction. In the presented QEC experiment, the error correction is implemented in a deterministic fashion due to the application of unconditional CV quantum entanglement^16^,^17^. A vacuum state and a squeezed vacuum state are utilized as the input states, respectively, to exhibit the QEC ability of the system for different input states. According to the standard notation for QEC code^1^, the presented five-wave-packet code should be expressed by [*n*, *k*, *d*] = [5, 1, 3], where *n* = 5 denote the number of used wave packets, *k* = 1 is the number of logical encoded input state, and *d* = 3 is the distance, which indicates how many errors can be tolerated, a code of distance *d* can correct up to (*d* − 1)/2 arbitrary errors at unspecified channels.

## Results

### Encoding

**Figure 1:**
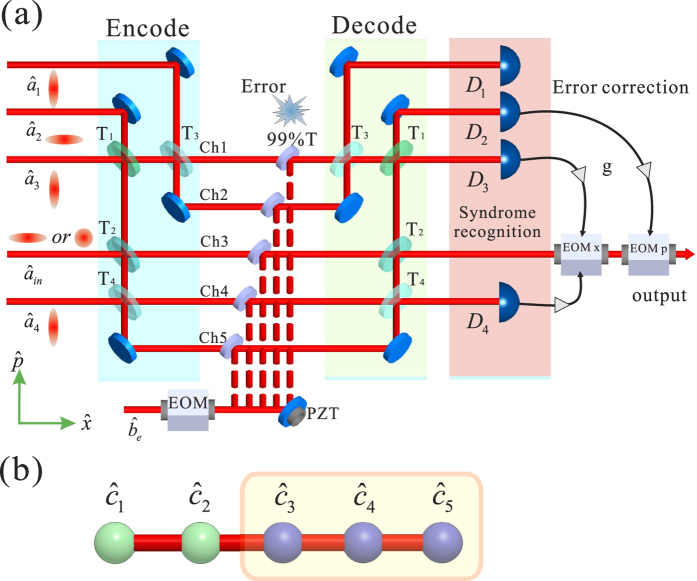
The schematic of the QEC scheme. (**a**) the schematic of experimental set-up. PZT: piezoelectric transducer. EOM: electro-optical modulator, T_1−4_: beam-splitters with 25%, 33%, 50%, and 50% transmission, respectively. Ch1-5: quantum channels. 99%T: a beam-splitter with 99% transmission. D_1_–D_4_: homodyne detectors, g: gain in the feedforward circuit. (**b**) the graph representation of the five-wave-packet code. The input state is encoded on submodes 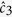, 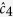 and 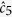 of a five-partite linear cluster state 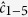.

The schematic of the CV QEC scheme is shown in Fig. 1(a). The QEC procedure contains five stages, which are encoding, error-in, decoding, syndrome recognition and correction, respectively. The encoding is completed by a beam-splitter network consisting of four beam-splitters (T_1_–T_4_). Four squeezed states with −3.5 dB squeezing 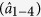 generated by three non-degenerate optical parametric amplifiers, are used as ancilla modes (see Supplementary Information for details). In the experiment, three amplitude-squeezed states, 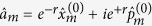 (*m* = 1, 3, 4), and a phase-squeezed state, 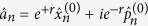 (*n* = 2) are applied, where *r* is the squeezing parameter (*r* = 0 and *r* = +∞ correspond to no squeezing and perfect squeezing, respectively), 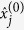 and 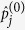 denote the amplitude and phase quadratures of the vacuum field, respectively. The transformation matrix of the encoding network is expressed by


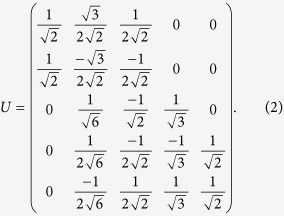


The unitary matrix can be decomposed by 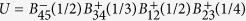. Here, 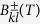 stands for the transformation of modes *k* and *l* on a beam-splitter, the corresponding transformation matrix is given by


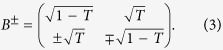


The input state 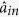 is encoded with the four ancilla modes by 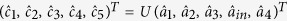. The encoded five modes are


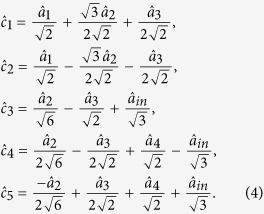


From equation (4) we can see, the input state is partially encoded on channels 3, 4 and 5 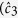, 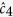 and 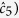 by means of the designed beam-splitter network, while the encoded states in channels 1 and 2 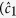 and 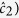 do not contain any information of the input state.

As shown in Fig. 1(b) the encoded five modes 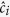 (*i* = 1, ..., 5) is the five submodes of a five-partite CV linear cluster entangled state^39^,^40^. The correlation noises of quadrature components among the encoded five wave-packets are expressed by 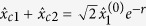, 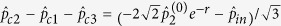, 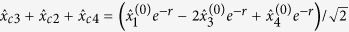, 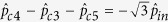, and 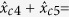 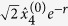. These expressions show that the correlation noises of 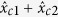, 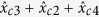 and 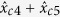 are smaller than the corresponding normalized shot-noise-level (SNL) for any non-zero squeezing of the ancilla modes. While the correlation noises of 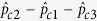 and 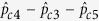 depend on the input state, i.e. they have different values for different input state. The inseparability criteria of the five-mode cluster entangled state are denoted by^41^


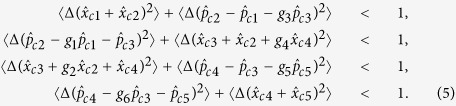


When all combinations of correlation variances on the left of the inequalities (5) are less than the normalized boundary on the right side, the five-wave-packet optical state is a CV cluster entangled state. With a vacuum input state and choosing the optimal gains of *g*_*i*_ (*i* = 1, 2...6) the inseparability criteria will be satisfied for any non-zero squeezing of the ancilla modes. In this case, the encoded five wave packets form a five-partite linear cluster entangled state.

### Error-in

The five encoded wave packets constitute five quantum channels, where the errors possibly occur. In the experiment, the noise is modulated on an excess optical beam 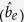 by an electro-optical modulator (EOM) drove by a sin-wave signal at 2 MHz to make an error beam firstly. Then, the error beam is randomly coupled into any one of the five coded wave packets each time by a mirror of 99% transmission. By sweeping the phase of the error wave packet with the piezoelectric translator (PZT) attached on a reflection mirror, a quasi-random displacement error is added on one of the five channels. The experimental operation corresponds to adding an error operator 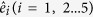 on a corresponding optical wave packet, the mathematic expression of which is 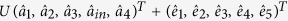, where only one of 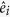 is non-zero when an error is occurring in one channel.

### Decoding

The decoding circuit is the inverse of the encoding circuit. After decoding, the output mode 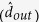 and syndrome modes 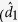, 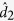, 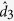 and 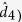 of the five channels are calculated by 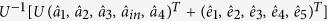. The decoded modes are


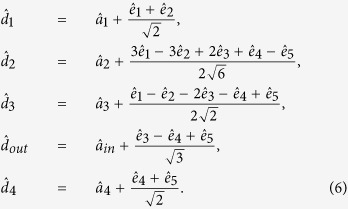


It is obvious that the input state and ancilla modes are recovered after the decoding stage and the errors are included in five output channels. Please note that the output state 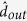 does not contain the errors 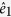 and 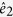, which means that the output state is immune from errors in channels 1 and 2. If the error occurs in channels 1 and 2, the output state will not be affected.

### Syndrome measurement

**Figure 2:**
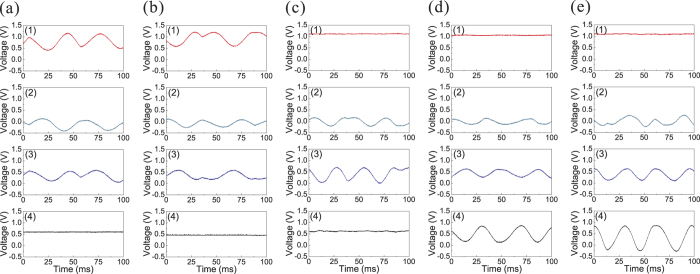
Error syndrome measurement results. (**a**–**e**) correspond to that a random displacement error is imposed on channel 1-5, respectively. The DC outputs of detectors D1-D4 are recorded by a four-channel digital oscilloscope and the results are shown in (1)–(4) from top to bottom, respectively.

From the decoded modes, we can see that the error in different channels results in different outputs of the homodyne detectors D_1_–D_4_. By the DC outputs of the homodyne detectors, we can determine in which channel the error is occurring (see Table 1). If a syndrome mode does not contain the error in a certain channel, the DC output of the corresponding detector will be a straight line without any fluctuation. When the error appearing in a syndrome mode, the DC output of the corresponding detector will be a line with fluctuation (coming from the error). A four-channel digital oscilloscope is used to record the DC output of detectors D_1_–D_4_. Figure 2 shows error syndrome measurement results. In Fig. 2(a), outputs with fluctuation are obtained by detectors D_1_, D_2_ and D_3_, and the fluctuations of detectors D_1_ and D_3_ are in-phase. The output of D_4_ is a straight line because the syndrome mode 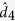 does not contain the error in channel 1 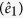. Comparing this result with Table 1, we can identify that an error is occurring in channel 1. In Fig. 2(b), we have outputs with fluctuation for detectors D_1_, D_2_ and D_3_, and the outputs of detectors D_1_ and D_3_ are out-of-phase, which means that an error is occurring in channel 2. With the same way, we know that the error occurs in channels 3, 4 and 5 from the measured results in Fig. 2(c–e), respectively.

### Error-correction

After the position of the error is identified, we can correct the error by feedfowarding the measurement results of the corresponding homodyne detectors D_1_–D_4_ to the output state with suitable gains (see Table 2). The partial encoding method simplifies the error correction procedure. When the error is occurring in channels 1 and 2, we do not need to correct it because it does not affect the output state. When the error occurs in the channel 3, 4 or 5, the output state will be stained by the error and we need to implement the feedforward of the measurement results.

**Figure 3:**
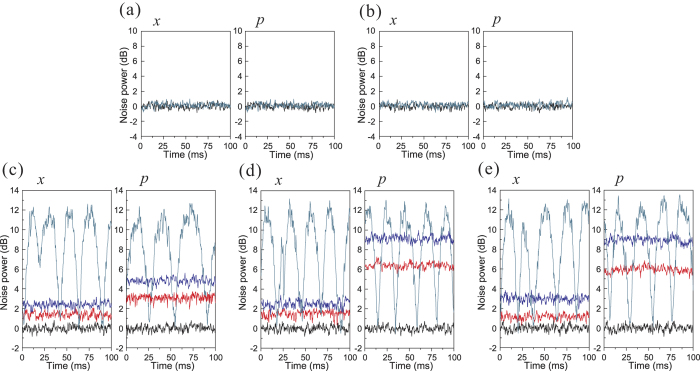
The error correction results for a vacuum input. (**a**–**e**) are the results of error correction with an error on channel 1–5, respectively. Black lines: the SNL. Cyan lines: the noises on amplitude (x) and phase (p) components of output state before error correction. Blue and red lines are the noises on x and p components of output state with the coherent and squeezed ancilla modes, respectively. Measurement frequency is 2 MHz, the spectrum analyzer resolution bandwidth is 30 kHz, and the video bandwidth is 300 Hz.

Figure 3 shows the results of QEC procedure for a vacuum input. The correction results for an error occurring in channels 1–5 are shown in Fig. 3(a–e), respectively. The quadrature components of output states before the error correction (cyan line), and after the correction (red and blue line) are given, where the red and blue lines correspond to the case using the squeezed and coherent state to be the ancilla modes, respectively, the black lines are the SNL. From Fig. 3(a,b), we can see that the output state is immune from errors appearing in channels 1 and 2. Thus, we do not need to perform error correction when errors are occurring in channels 1 and 2. When the error is imposed on channels 3, 4 and 5, the output state contains the error signal before the error correction [cyan lines in Fig. 3(c–e)]. In the error correction procedure, the measurement results of detectors 3 (or 4) and 2 are fedforward to the output state (see Table 2). Figure 3(c–e) show, when the squeezed ancilla modes are utilized, the noises on the output state are reduced. The better the squeezing, the lower the noise of output state. When the used ancilla modes are perfect squeezed states, the output state will totally overlap with the input vacuum state. The measured noise power of the output state can be found in Supplementary Information.

**Figure 4:**
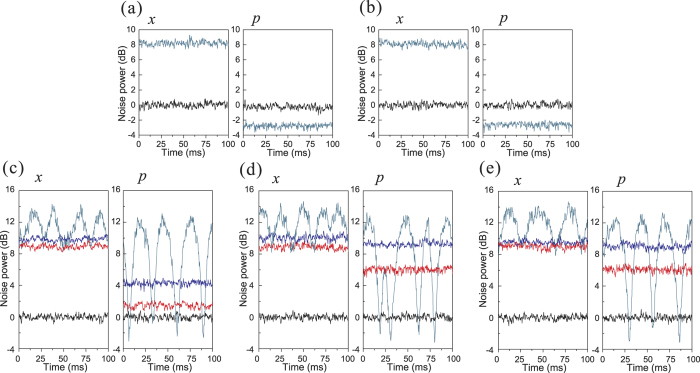
The error correction results for a phase-squeezed input. (**a**–**e**) are the results of error correction with an error on channel 1–5, respectively. Black lines: the SNL. Cyan lines: the noises of the amplitude (x) and phase (p) components of output state before the error correction. Blue and red lines correspond to the noises levels of output state after the error correction with the coherent and squeezed ancilla modes, respectively. Measurement frequency is 2 MHz, the spectrum analyzer resolution bandwidth is 30 kHz, and the video bandwidth is 300 Hz.

QEC results with a phase-squeezed state (−3.5 dB/8.9 dB squeezing/antisqueezing) as the input state are shown in Fig. 4. Figure 4(a–e) are the results of the corrections for an error in channels 1–5, respectively. In Fig. 4(a,b), the output state is still a phase squeezed state before the error correction (cyan line) when errors are occurring in channels 1 and 2, which shows that the output state is not affected by errors in channels 1 and 2. The measured squeezing and antisqueezing of the output state are −2.78 dB/8.22 dB and −2.73 dB/8.09 dB for the errors in channels 1 and 2, respectively. The decrease of the squeezing derives from the imperfection in the experiment, such as channel loss and fluctuation of phase locking system. When the error is imposed on channel 3, 4 and 5, the output state becomes very noisy before error correction (cyan line). After error correction, the measured noise of the output state with the squeezed ancilla modes (red line) is below that using coherent states as the ancilla modes (blue line).

The fidelity 
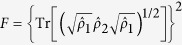
, which denotes the overlap between the experimentally obtained output state 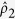 and the input state 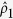, is utilized to quantify the performance of the QEC code. The fidelity for two Gaussian states 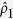 and 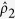 with the covariance matrices *σ*_*j*_ is expressed by^42^,^43^


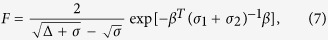


where Δ = det(*σ*_1_ + *σ*_2_), *σ* = (det* σ*_1_ − 1)(det* σ*_2_ − 1), *β* = *α*_2_ − *α*_1_, and *α*_*j*_ is the mean amplitudes *α*_*j*_ ≡ (*α*_*jx*_, *α*_*jp*_)^*T*^ (*j* = 1, 2), *σ*_1_ and *σ*_2_ are the covariance matrices for the input state 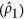 and the experimentally obtained output state 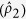, respectively. In our experiment, a vacuum state and a squeezed vacuum state are used for the input states, respectively, and the mean amplitude for the both states equals to zero. If squeezed states with infinite squeezing (*r* → ∞) are utilized as the ancilla states, the fidelity will equal to 1. When all ancilla modes are the coherent states of light with zero classical noise (*r* = 0), the obtained fidelity of the output state is the corresponding classical limit^31^,^32^. Since the errors in channels 1 and 2 do not affect the output state, the obtained fidelity is near unity (0.99). The fidelity obtained with squeezed states to be the ancilla modes is higher than that obtained with coherent states when error appears in channel 3, 4 and 5 (see Table 2).

## Discussion

The presented compact five-wave-packet QEC code can be applied to correct a single stochastic error in a single quantum channel. For this type of error correction one usually assume that errors occur stochastically with a small probability so that multiple errors are unlikely to happen. When two or more errors are occurring simultaneously on the encoded channels, the errors can not be identified and corrected because the syndrome measurement will be confusing^31^,^32^.

The general error 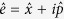 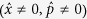 and *x*-displacement error 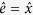 can be well recognized and corrected suitably with the presented scheme. For the *p*-displacement error 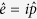, it is unclear which channel the error comes from since only the phase measurement of detector D_2_ has output with fluctuation for all five channels (see Table 1). If this happens in the syndrome measurement results, we need to apply a Fourier transformation *F* (a 90° rotation in the phase space) on each ancilla mode in the encoding stage. In this way, the output state is given by 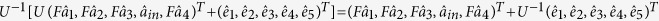 and thus in the syndrome stage, the amplitude quadrature of detector D_2_ and phase quadratures of detectors D_1_, D_3_, D_4_ are measured. Such that, the *p*-displacement error can be identified by the outputs with fluctuation from detectors D_1_, D_3_ and D_4_.

In summary, we experimentally demonstrated a compact five-wave-packet CV QEC code using a five-partite cluster entangled state of light. The QEC code is implemented only with linear optics operations and four ancilla squeezed states of light. The compact optics circuit can increase data rates and decrease chance of further error occurring. The presented partial encoding method may simplify the error correction procedure and improve the efficiency of QEC. The presented experiment is the first experimental demonstration of the approach proposed by S. L. Braunstein and T. A. Walker for designing linear optics circuits of CV QEC code, which has potential application in constructing future CV quantum information networks.

## Additional Information

**How to cite this article**: Hao, S. *et al*. Five-wave-packet quantum error correction based on continuous-variable cluster entanglement. *Sci. Rep*. **5**, 15462; doi: 10.1038/srep15462 (2015).

## Supplementary Material

Supplementary Information

## Figures and Tables

**Table 1:** Error syndrome measurements.

The error channel	Detectors with fluctuation	Measurement basis
1	1, 3 (in-phase)	x
	2	p
2	1, 3 (out-of-phase)	x
	2	p
3	3	x
	2	p
4	3, 4 (out-of-phase)	x
	2	p
5	3, 4 (in-phase)	x
	2	p

**Table 2:** Error correction feedforward components and the obtained fidelities.

Error in channel	Quadrature of output	Feedforward components	Fidelity with coherent state	Fidelity with squeezing
1	x	0	0.99 (0.99)	0.99 (0.99)
	p	0		
2	x	0	0.99 (0.99)	0.99 (0.99)
	p	0		
3	x	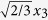	0.60 (0.68)	0.75 (0.85)
	p	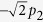		
4	x	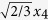	0.40 (0.42)	0.56 (0.60)
	p	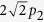		
5	x	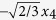	0.39 (0.44)	0.59 (0.59)
	p	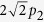		

Fidelities in and out of brackets are for the case of a squeezed and a vacuum state used as input state, respectively.
