# Supplementary material for: Five-wave-packet quantum error correction based on continuous-variable cluster entanglement
Source: Sci Rep. 2015 Oct 26;5:15462. doi: 10.1038/srep15462 (PMC4620507; doi:10.1038/srep15462)
Supplement: Supplementary Information [file srep15462-s1.pdf]

Supplementary Information for "Five-wave-packet quantum error correction based on continuous-variable cluster entanglement"

Shuhong Hao, Xiaolong Su,* and Caixing Tian, Changde Xie and Kunchi Peng

State Key Laboratory of Quantum Optics and Quantum Optics Devices,

Institute of Opto-Electronics, Shanxi University,

Taiyuan, 030006, People's Republic of China

I. EXPERIMENTAL DETAILS

The amplitude-squeezed and phase-squeezed states are produced by three non-degenerate optical parametric amplifiers (NOPAs) with identical configuration. These NOPAs are pumped by a common laser source, which is a continuous wave intracavity frequency-doubled and frequency-stabilized Nd:YAP/LBO(Nd-doped $YAlO_3$ perorskite/lithium triborate) laser [1]. Each of NOPAs consists of an α-cut type-II KTP crystal and a concave mirror [2]. The front face of the KTP is coated to be used for the input coupler and the concave mirror serves as the output coupler of the squeezed states. The transmissions of the input coupler at 540 nm and 1080 nm are 99.8% and 0.04%, respectively. The transmissions of the output coupler at 540 nm and 1080 nm are 0.5% and 5.2%, respectively. An NOPA simultaneously generates an amplitude-squeezed state and a phase-squeezed state in two orthogonal polarizations [3]. The ancilla modes $\hat{a}_1$, $\hat{a}_2$ and $\hat{a}_3$, $\hat{a}_4$ and the phase-squeezed input state, are generated by three NOPAs respectively. Three NOPAs are locked individually by using Pound-Drever-Hall method with a phase modulation of 56 MHz on 1080 nm laser beam. All NOPAs are operated at deamplification condition, which corresponds to lock the relative phase between the pump laser and the injected signal to (2n+1)π (n is the integer).

The transmission efficiency of an optical beam from NOPA to a homodyne detector is around 96%. The quantum efficiency of a photodiode (FD500W-1064, Fermionics) used in the homodyne detection system is 95%. The interference efficiency on a beam-splitter is about 99%.

*Electronic address: suxl@sxu.edu.cn

The Fourier transformation F needed for the correction of p-displacement error is a 90° rotation in the phase space, which changes the squeezing direction of the squeezed state. The Fourier transformations on the ancilla modes $\hat{a}_1$ and $\hat{a}_4$ can be completed by changing the relative phase difference on the beam-splitters T_3 and T_4 from 0 to $\pi/2$, respectively. The Fourier transformations on the ancilla modes $\hat{a}_2$ and $\hat{a}_3$ can be implemented by exchanging the position of $\hat{a}_2$ and $\hat{a}_3$ on the beam-splitter T_1, which can be simply achieved by rotating the half wave-plate for 45° placed at the output port of the NOPA that is because $\hat{a}_2$ and $\hat{a}_3$ are produced from one NOPA [3].

II. DETAILS OF SYNDROME AND ERROR-CORRECTION PROCEDURE

When the error is occurring in channel 1, we have non-zero syndrome measurement for

$$\begin{aligned}
\hat{x}_{_{D1}} &= \frac{\hat{x}_{e_1}}{\sqrt{2}} + \hat{x}_1^{(0)} e^{-r}, \\
\hat{x}_{_{D3}} &= \frac{\hat{x}_{e_1}}{2\sqrt{2}} + \hat{x}_3^{(0)} e^{-r}, \\
\hat{p}_{_{D2}} &= \frac{3\hat{p}_{e_1}}{2\sqrt{6}} + \hat{p}_2^{(0)} e^{-r},
\end{aligned} \tag{1}$$

where the outputs of $\hat{x}_{_{D1}}$ and $\hat{x}_{_{D3}}$ are in-phase. At this case, the output state is

$$\begin{aligned}
\hat{x}_{out} &= \hat{x}_{in}, \\
\hat{p}_{out} &= \hat{p}_{in},
\end{aligned} \tag{2}$$

which is immune from the error in channel 1, thus we do not need any correction.

When the error is occurring in channel 2, non-zero syndrome measurement is obtained for

$$\begin{aligned}
\hat{x}_{_{D1}} &= \frac{\hat{x}_{e_2}}{\sqrt{2}} + \hat{x}_1^{(0)} e^{-r}, \\
\hat{x}_{_{D3}} &= \frac{-\hat{x}_{e_2}}{2\sqrt{2}} + \hat{x}_3^{(0)} e^{-r}, \\
\hat{p}_{_{D2}} &= \frac{-3\hat{p}_{e_2}}{2\sqrt{6}} + \hat{p}_2^{(0)} e^{-r},
\end{aligned} \tag{3}$$

where $\hat{x}_{_{D1}}$ and $\hat{x}_{_{D3}}$ are out-of-phase. The corresponding output state is

$$\begin{aligned}
\hat{x}_{out} &= \hat{x}_{in}, \\
\hat{p}_{out} &= \hat{p}_{in},
\end{aligned} \tag{4}$$

and we do not need any correction.

When the error is occurring in channel 3, non-zero syndrome measurements are obtained for

$$\begin{aligned} \hat{x}_{D3} &= \frac{-2\hat{x}_{e3}}{2\sqrt{2}} + \hat{x}_3^{(0)}e^{-r}, \\ \hat{p}_{D2} &= \frac{2\hat{p}_{e3}}{2\sqrt{6}} + \hat{p}_2^{(0)}e^{-r}. \end{aligned} \tag{5}$$

In this case, the output state is

$$\begin{aligned} \hat{x}_{out} &= \hat{x}_{in} + \frac{\hat{x}_{e_3}}{\sqrt{3}}, \\ \hat{p}_{out} &= \hat{p}_{in} + \frac{\hat{p}_{e_3}}{\sqrt{3}}. \end{aligned} \tag{6}$$

To eliminate the error, $\frac{\sqrt{2}}{\sqrt{3}}\hat{x}_{D3}$ and $-\sqrt{2}\hat{p}_{D2}$ should be fedforward to $\hat{x}_{out}$ and $\hat{p}_{out}$, respectively. The corrected output mode is given by

$$\begin{aligned} \hat{x}'_{out} &= \hat{x}_{in} + \frac{\sqrt{2}}{\sqrt{3}}\hat{x}_3^{(0)}e^{-r}, \\ \hat{p}'_{out} &= \hat{p}_{in} - \sqrt{2}\hat{p}_2^{(0)}e^{-r}. \end{aligned} \tag{7}$$

The noise powers of the output state are

$$\left\langle \Delta^2 \hat{x}'_{out} \right\rangle = \left\langle \Delta^2 \hat{x}_{in} \right\rangle + \frac{2}{3} \times \frac{1}{4}e^{-2r} \tag{8}$$

and

$$\left\langle \Delta^2 \hat{p}'_{out} \right\rangle = \left\langle \Delta^2 \hat{p}_{in} \right\rangle + 2 \times \frac{1}{4}e^{-2r}, \tag{9}$$

respectively.

When the error is occurring in channel 4, we have non-zero syndrome measurements on

$$\begin{aligned} \hat{x}_{D3} &= \frac{-\hat{x}_{e_4}}{2\sqrt{2}} + \hat{x}_3^{(0)}e^{-r}, \\ \hat{x}_{D4} &= \frac{\hat{x}_{e_4}}{\sqrt{2}} + \hat{x}_4^{(0)}e^{-r}, \\ \hat{p}_{D2} &= \frac{\hat{p}_{e_4}}{2\sqrt{6}} + \hat{p}_2^{(0)}e^{-r}, \end{aligned} \tag{10}$$

where $\hat{x}_{D3}$ and $\hat{x}_{D4}$ are out-of-phase. The corresponding output state is

$$\begin{aligned} \hat{x}_{out} &= \hat{x}_{in} - \frac{\hat{x}_{e4}}{\sqrt{3}}, \\ \hat{p}_{out} &= \hat{p}_{in} - \frac{\hat{p}_{e4}}{\sqrt{3}}. \end{aligned} \tag{11}$$

The measurement results of $\frac{\sqrt{2}}{\sqrt{3}}\hat{x}_{D4}$ and $2\sqrt{2}\hat{p}_{D2}$ should be fedforward to $\hat{x}_{out}$ and $\hat{p}_{out}$ to eliminate the error. The corrected output mode is

$$\begin{aligned}\hat{x}'_{out} &= \hat{x}_{in} + \frac{\sqrt{2}}{\sqrt{3}}\hat{x}_4^{(0)}e^{-r},\\ \hat{p}'_{out} &= \hat{p}_{in} + 2\sqrt{2}\hat{p}_2^{(0)}e^{-r},\end{aligned} \tag{12}$$

and the corresponding noise powers are

$$\left\langle \Delta^2 \hat{x}'_{out} \right\rangle = \left\langle \Delta^2 \hat{x}_{in} \right\rangle + \frac{2}{3} \times \frac{1}{4}e^{-2r}, \tag{13}$$

and

$$\left\langle \Delta^2 \hat{p}'_{out} \right\rangle = \left\langle \Delta^2 \hat{p}_{in} \right\rangle + 8 \times \frac{1}{4}e^{-2r}, \tag{14}$$

respectively.

When the error is occurring in channel 5, we have non-zero syndrome measurements on

$$\begin{aligned}\hat{x}_{D3} &= \frac{\hat{x}_{e5}}{2\sqrt{2}} + \hat{x}_3^{(0)}e^{-r},\\ \hat{x}_{D4} &= \frac{\hat{x}_{e_5}}{\sqrt{2}} + \hat{x}_4^{(0)}e^{-r},\\ \hat{p}_{D2} &= \frac{-\hat{p}_{e5}}{2\sqrt{6}} + \hat{p}_2^{(0)}e^{-r},\end{aligned} \tag{15}$$

where $\hat{x}_{D3}$ and $\hat{x}_{D4}$ are in-phase. The output state is

$$\begin{aligned}\hat{x}_{out} &= \hat{x}_{in} + \frac{\hat{x}_{e_5}}{\sqrt{3}},\\ \hat{p}_{out} &= \hat{p}_{in} + \frac{\hat{p}_{e_5}}{\sqrt{3}}.\end{aligned} \tag{16}$$

By feedforwarding $\frac{-\sqrt{2}}{\sqrt{3}}\hat{x}_{D4}$ and $2\sqrt{2}\hat{p}_{D2}$ to $\hat{x}_{out}$ and $\hat{p}_{out}$, the error will be corrected. The output mode after correction is

$$\begin{aligned}\hat{x}'_{out} &= \hat{x}_{in} - \frac{\sqrt{2}}{\sqrt{3}}\hat{x}_4^{(0)}e^{-r},\\ \hat{p}'_{out} &= \hat{p}_{in} + 2\sqrt{2}\hat{p}_2^{(0)}e^{-r}\end{aligned} \tag{17}$$

and the corresponding noise powers are

$$\left\langle \Delta^2 \hat{x}'_{out} \right\rangle = \left\langle \Delta^2 \hat{x}_{in} \right\rangle + \frac{2}{3} \times \frac{1}{4}e^{-2r}, \tag{18}$$

and

$$\left\langle \Delta^2 \hat{p}'_{out} \right\rangle = \left\langle \Delta^2 \hat{p}_{in} \right\rangle + 8 \times \frac{1}{4}e^{-2r}, \tag{19}$$

respectively.

Table S1 The noise powers of the output state (with the unit of dB).

Error in channel	Quadrature of output	Noise of the output state without squeezing on ancilla modes	Noise of the output state with squeezing on ancilla modes
1	x	0.15 ± 0.30 (8.22 ± 0.31)	
	p	0.13 ± 0.30 (-2.78 ± 0.27)	
2	x	0.19 ± 0.29 (8.09 ± 0.31)	
	p	0.18 ± 0.30 (-2.73 ± 0.29)	
3	x	2.39 ± 0.28 (9.85 ± 0.27)	1.37 ± 0.29 (8.93 ± 0.27)
	p	4.80 ± 0.29 (4.28 ± 0.28)	3.07 ± 0.31 (1.46 ± 0.30)
4	x	2.47 ± 0.34 (9.96 ± 0.32)	1.49 ± 0.29 (8.89 ± 0.29)
	p	9.13 ± 0.30 (9.25 ± 0.27)	6.40 ± 0.28 (6.04 ± 0.30)
5	x	2.99 ± 0.31 (9.51 ± 0.27)	1.14 ± 0.28 (9.02 ± 0.30)
	p	9.01 ± 0.32 (9.03 ± 0.32)	5.94 ± 0.30 (6.10 ± 0.33)

The noise powers of the output state in and out of brackets are for the case of a squeezed and a vacuum state used as input state, respectively.

III. NOISE POWER OF THE OUTPUT STATE

The measured noise power of the output state in QEC is shown in table S1. Measurement frequency of noise power is 2 MHz, the spectrum analyzer resolution bandwidth is 30 kHz, and the video bandwidth is 300 Hz.

[1] Y. Wang, Y. Zheng, C. Xie, and K. Peng, IEEE J. Quantum Electronics **47**, 1006-1013 (2011).

[2] Y. Wang, H. Shen, X. Jin, X. Su, C. Xie, and K. Peng, Opt. Express **18**, 6149-6155 (2010).

[3] Y. Zhang, H. Wang, X. Li, J. Jing, C. Xie, and K. Peng, Phys. Rev. A **62**, 023813 (2000).
